# Supplementary material for: Single cell transcriptomic profiling identifies tumor-acquired and therapy-resistant cell states in pediatric rhabdomyosarcoma
Source: Nat Commun. 2024 Jul 26;15:6307. doi: 10.1038/s41467-024-50527-2 (PMC11282092; doi:10.1038/s41467-024-50527-2)
Supplement: Supplementary file 1 — Supplementary Information [file 41467_2024_50527_MOESM1_ESM.pdf]

SUPPLEMENTARY FIGURES AND LEGENDS

Single cell transcriptomic profiling identifies tumor-acquired and therapy-resistant cell states in pediatric rhabdomyosarcoma

Sara G Danielli[1*], Yun Wei[2,3,4*], Michael A Dyer[5], Elizabeth Stewart[5,6], Heather Sheppard[7], Marco Wachtel[1#], Beat W Schäfer[1#], Anand G Patel[5,6#], David M Langenau[2,3,4#]

[1]Department of Oncology and Children's Research Center, University Children's Hospital of Zurich, Steinwiesstrasse 75, 8032 Zürich, Switzerland.

[2]Molecular Pathology Unit, Massachusetts General Research Institute, Charlestown, MA, USA, 02129.

[3]Krantz Family Center for Cancer Research, Massachusetts General Hospital, Charlestown, MA, 02129, USA.

[4]Harvard Stem Cell Institute, Cambridge, MA, USA.

[5]Department of Developmental Neurobiology, St. Jude Children's Research Hospital, Memphis, TN 38105, USA.

[6]Department of Oncology, St. Jude Children's Research Hospital, Memphis, TN 38105, USA.

[7]Department of Pathology, St. Jude Children's Research Hospital, Memphis, TN 38105, USA.

* These authors contributed equally

# These authors jointly supervised this work

Corresponding authors:

David M. Langenau, dlangenau@mgh.harvard.edu

Anand G. Patel, anand.patel2@stjude.org

Beat W. Schäfer, beat.schaefer@kispi.uzh.ch

Marco Wachtel. marco.wachtel@kispi.uzh.ch

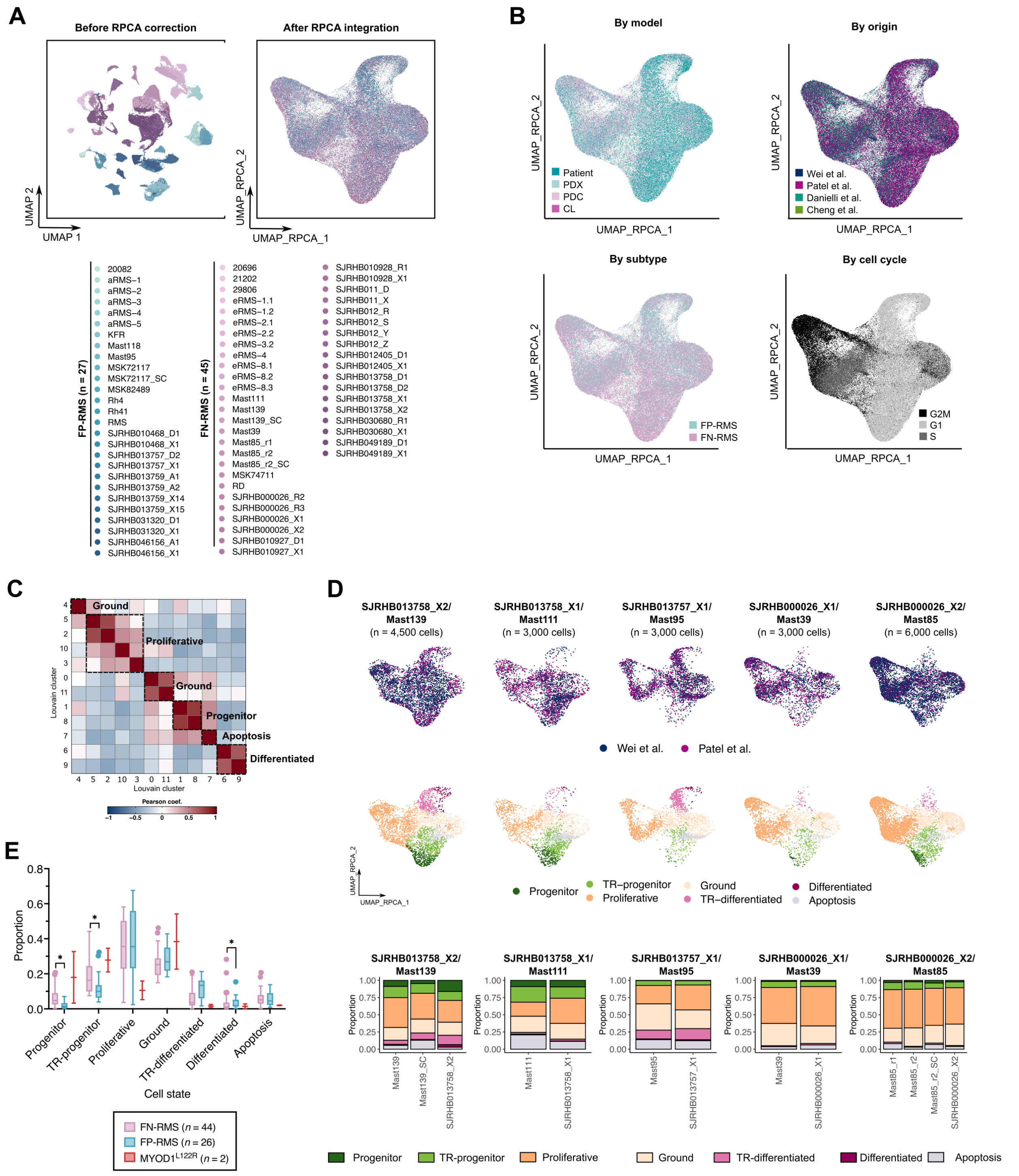

Fig. S1. Integrated analysis of single cell and single nuclei sequencing of human RMS. A) UMAP rendering of RMS cells before (left) and after RPCA integration (right). Cells are colored based on sample as noted in the legend below the PCA plot. "_SC",

single-cell derived PDX sample, "_D", diagnostic, "_R", recurrence, "_A", autopsy, "_X". xenograft. **B)** UMAP rendering of RMS cells colored by model, publication of origin, RMS subtype, or cell cycle. **C)** Heatmap showing correlation matrix similarities of Louvain clusters identified by integrated analysis of all RMS samples together. Clusters were combined based on gene expression similarity and cell states are noted by dashed lines. **D)** Comparison of relative cell state constitution across five PDX models sequenced across different publications. UMAP plots (top) and bar plots showing cell state composition (bottom). **E)** Compositional changes calculated with scCODA between different RMS molecular subtypes. Asterisks denote statistically credible differences in composition with a false discovery rate of $\leq$ 0.05.

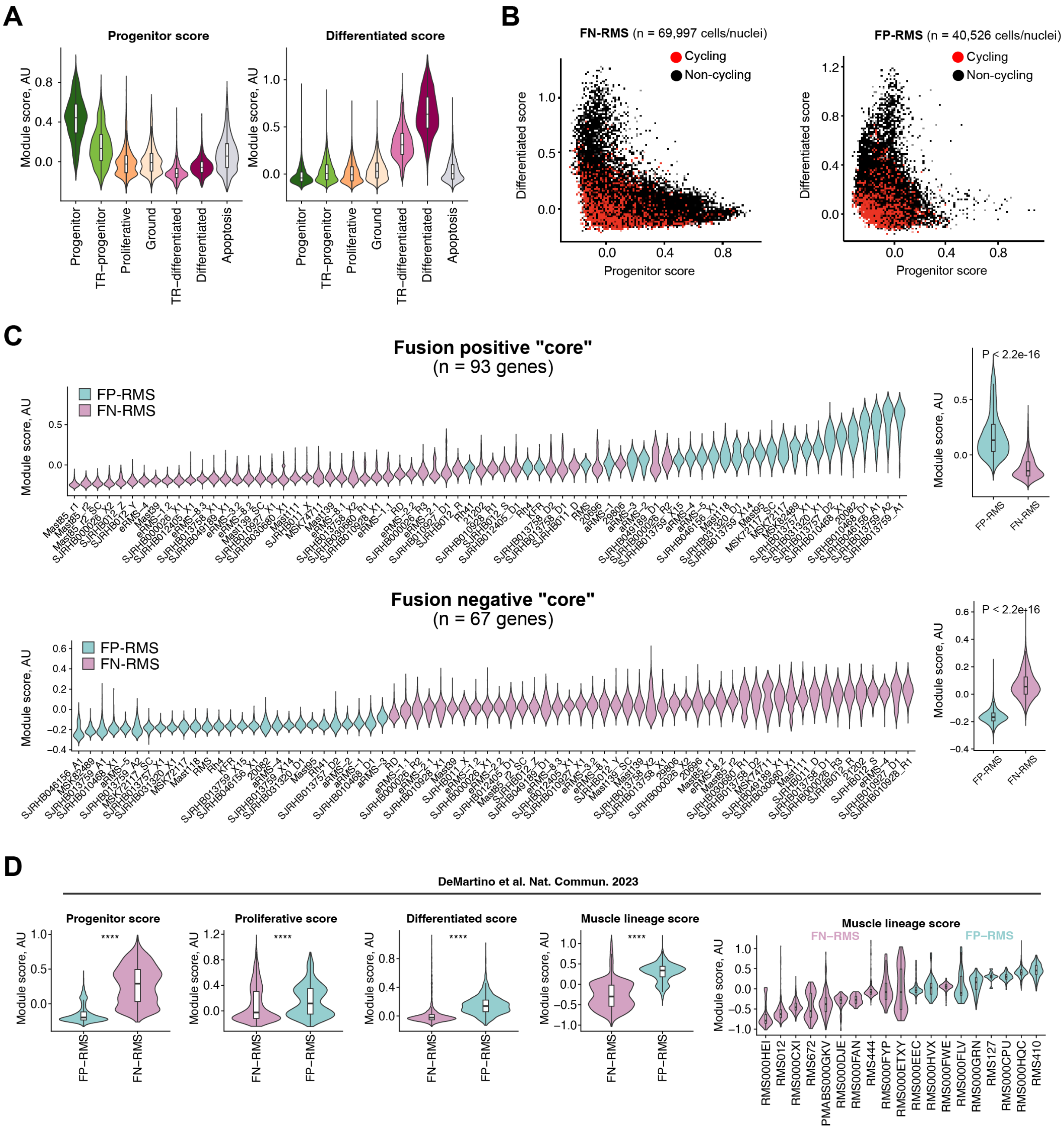

Fig. S2. Gene expression across single cells from FN- and FP-RMS. A) Violin plots showing module expression of progenitor and differentiated scores across different tumor cell states identified in the combined RMS atlas. **B)** Scatter plots comparing individual cell expression of progenitor and differentiated metaprograms across FN-RMS (left) or FP-RMS samples (right). Cells/nuclei are colored by cycling status. **C)** Violin plots showing

expression of core-signature gene profiles that distinguish the two major RMS subtypes as defined by Wei et al. . Left: Analysis of each tumor model. Right: Summary analysis after combining all samples together. **D)** Violin plots showing expression of RMS-specific module scores (progenitor, proliferative, differentiated and muscle lineage) across FN-RMS and FP-RMS samples reanalyzed from DeMartino et al. [1] The muscle lineage score of individual samples, ordered by increasing score, is shown on the right. Statistical analysis used Student's T-test comparison with p-values noted. AU, arbitrary unit.

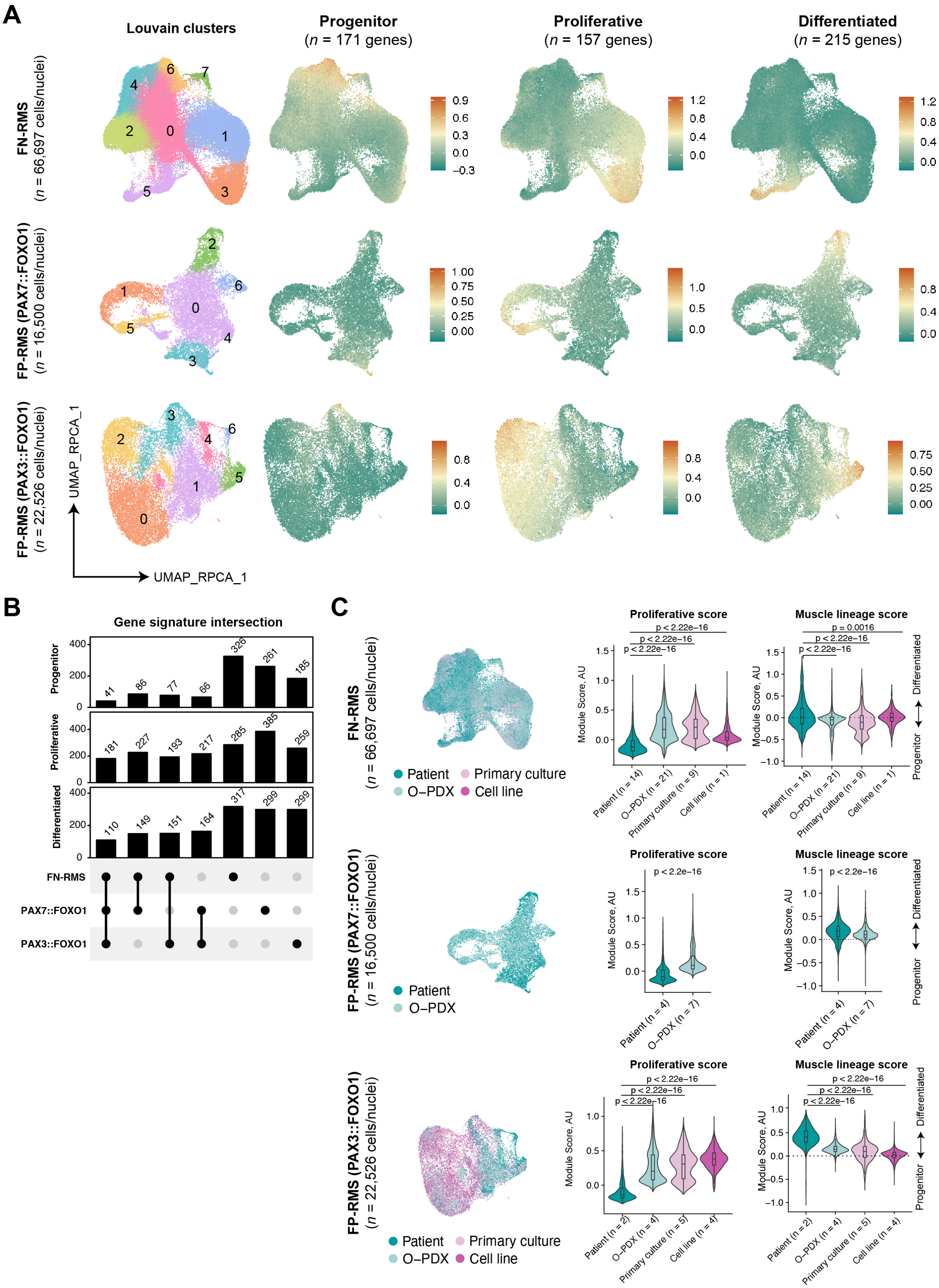
A
Louvain clusters
Progenitor
(n = 171 genes)
Proliferative
(n = 157 genes)
Differentiated
(n = 215 genes)
FN-RMS
(n = 66,697 cells/nuclei)
FP-RMS (PAX7::FOXO1)
(n = 16,500 cells/nuclei)
FP-RMS (PAX3::FOXO1)
(n = 22,526 cells/nuclei)
UMAP_RPCA_1
UMAP_RPCA_1
B
Gene signature intersection
Progenitor
Proliferative
Differentiated
FN-RMS
PAX7::FOXO1
PAX3::FOXO1
C
Patient
O–PDX
Primary culture
Cell line
Proliferative score
Muscle lineage score
Module Score, AU
Differentiated
Progenitor
Patient (n = 14)
O–PDX (n = 21)
Primary culture (n = 9)
Cell line (n = 1)
Patient (n = 4)
O–PDX (n = 7)
Patient (n = 2)
O–PDX (n = 4)
Primary culture (n = 5)
Cell line (n = 4)

Fig. S3. Tripartite muscle intra-tumoral heterogeneity is largely retained across patient and preclinical RMS models. A) UMAP renderings of FN-RMS, PAX7::FOXO1 and PAX3::FOXO1 FP-RMS. Cells/nuclei were integrated independently and colored based on Louvain clusters (left) or by the expression of cell state metaprograms defined in this publication (right). **B**) Upset plot of the shared marker genes for the progenitor, proliferative and differentiated signatures derived from the subtype-specific analysis. The progenitor signature shows little overlap among the three RMS subtypes (only 41 genes), whereas the proliferative and differentiated signatures share a higher fraction of overlapping genes across models. **C)** Analysis of tripartite muscle cell states in patient tissue, orthotopic xenografts, primary cultures, or established cell lines. UMAP plots comparing RMS subtype (left) and Violin plot quantitation (right). Adjusted *p*-values calculated by one-way ANOVA (FN-RMS and PAX3::FOXO1 FP-RMS) or by Student's T-test (PAX7::FOXO1 FP-RMS).

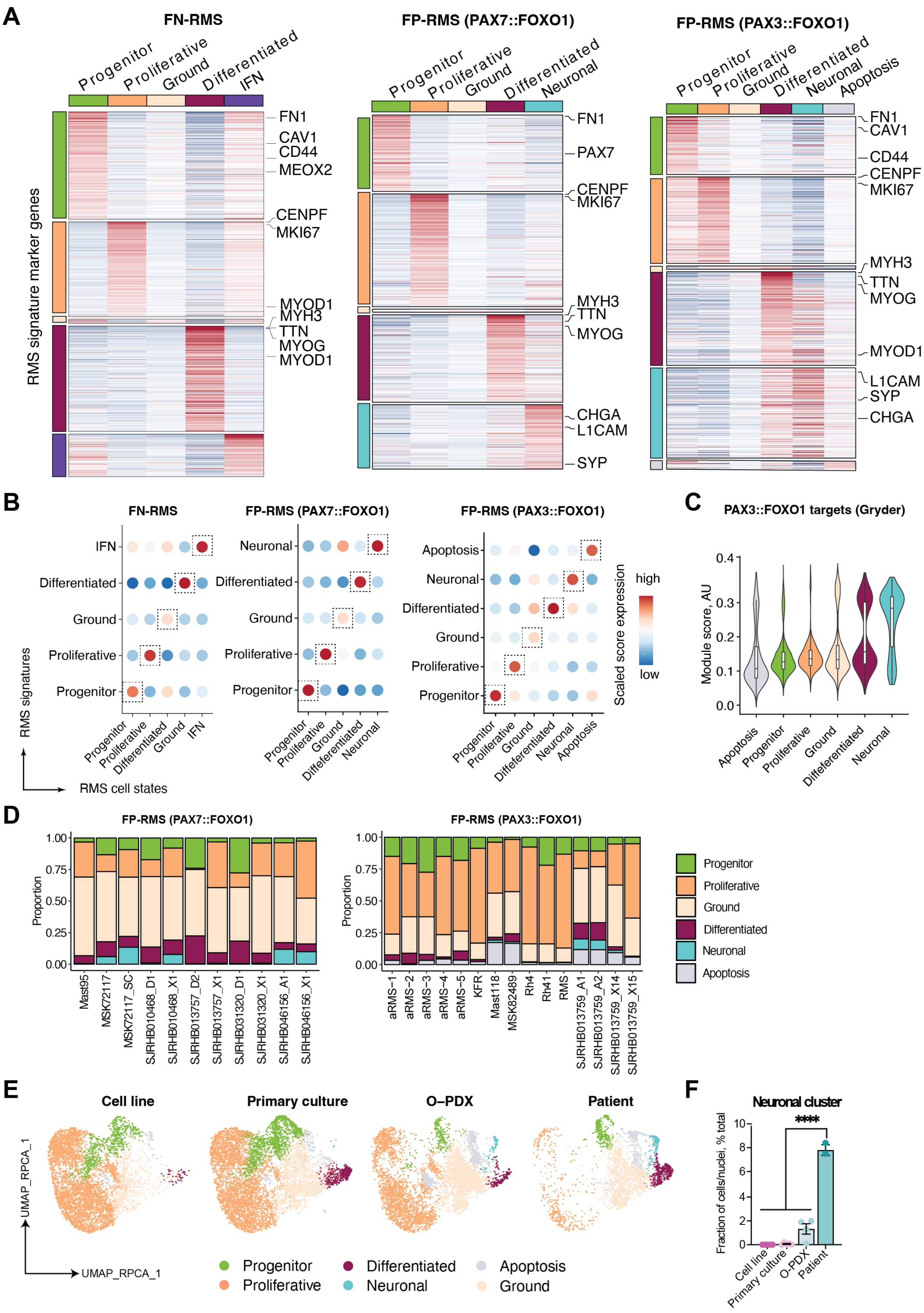
A
FN-RMS
FP-RMS (PAX7::FOXO1)
FP-RMS (PAX3::FOXO1)
Progenitor
Proliferative
Ground
Differentiated
IFN
Neuronal
Apoptosis
RMS signature marker genes
FN1
CAV1
CD44
MEOX2
CENPF
MKI67
MYOD1
MYH3
TTN
MYOG
PAX7
CHGA
L1CAM
SYP
B
RMS signatures
RMS cell states
Scaled score expression
high
low
C
PAX3::FOXO1 targets (Gryder)
Module score, AU
0.0
0.1
0.2
0.3
0.4
D
Proportion
0.00
0.25
0.50
0.75
1.00
Mast95
MSK72117
MSK72117_SC
SJRHB010468_D1
SJRHB010468_X1
SJRHB013757_D2
SJRHB013757_X1
SJRHB031320_D1
SJRHB031320_X1
SJRHB046156_A1
SJRHB046156_X1
aRMS−1
aRMS−2
aRMS−3
aRMS−4
aRMS−5
KFR
Mast118
MSK82489
Rh4
Rh41
RMS
SJRHB013759_A1
SJRHB013759_A2
SJRHB013759_X14
SJRHB013759_X15
E
Cell line
Primary culture
O−PDX
Patient
UMAP_RPCA_1
UMAP_RPCA_1
F
Neuronal cluster
Fraction of cells/nuclei, % total
0
2
4
6
8
10
****
Cell line
Primary culture
O-PDX
Patient

Fig. S4. Neuronal cell states are not retained in FP-RMS cell line and primary cultures. A) Heatmap showing expression of the marker genes identified across tumor cell states. Selected genes are displayed on the right. **B)** Dotplot showing expression of tumor signatures across the identified cell states. Cells across each state were scored for the top 50 genes identified for each RMS signature. Boxes denote highest scoring correlations between assigned signature and cell state. **C)** Violin plot showing expression of the PAX3::FOXO1 target gene signature identified in Gryder et al. [2] across the identified FP-RMS (PAX3::FOXO1) tumor cell states, ordered by increasing scores. **D)** Barplot showing the proportion of RMS cell states across each sample. **E)** UMAP plots of integrated PAX3::FOXO1 FP-RMS samples colored by cell states and analyzed across tumor models **F)** Quantification of the neuronal cluster fraction across PAX3::FOXO1 FP-RMS samples. Ordinary one-way analysis of variance (ANOVA) with Dunnett's multiple comparison correction. ****P ≤ 0.0001).

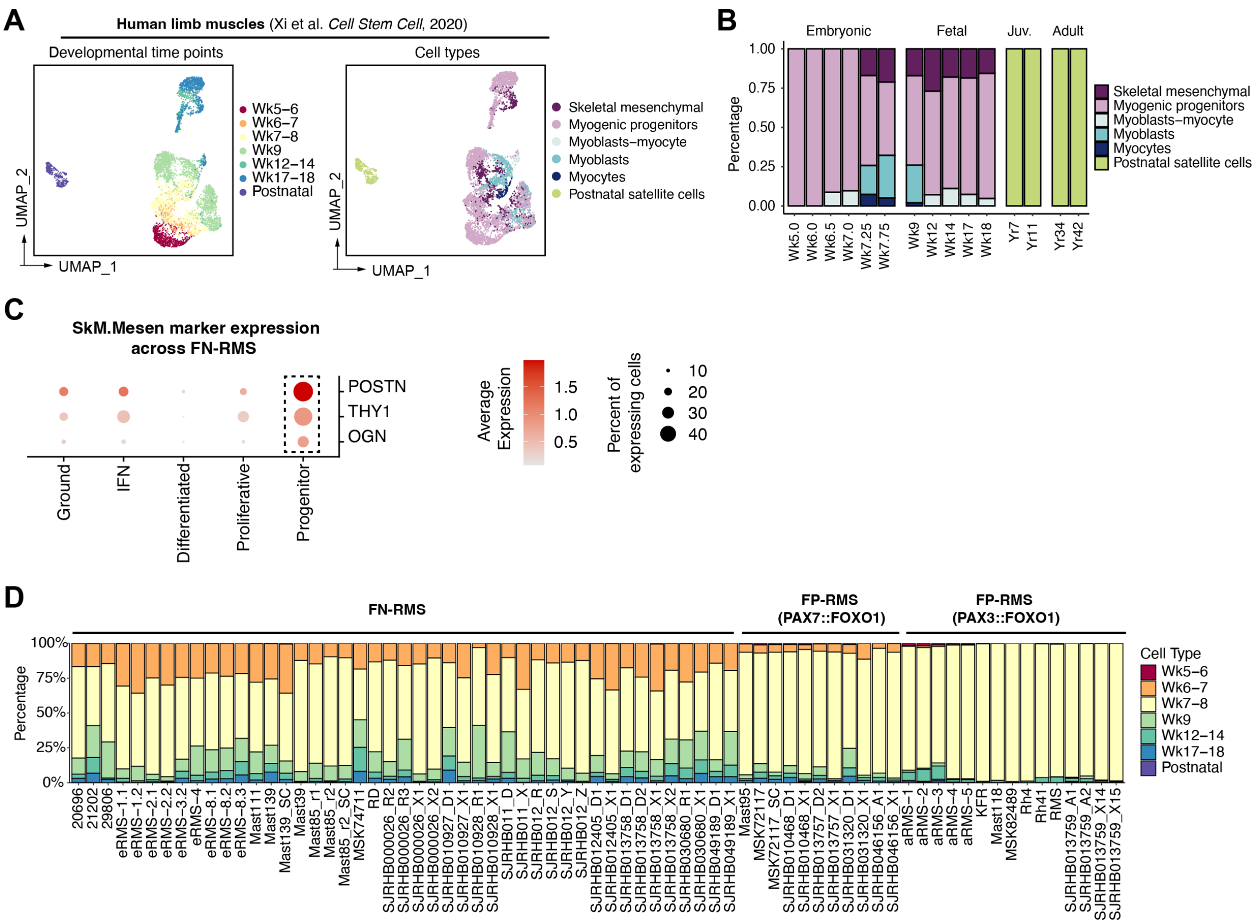

Fig. S5: Mapping RMS cells to human skeletal muscle development. A) UMAP plots of cells profiled during human skeletal muscle development in Xi et al. [3] and reanalyzed in this publication. Cells are colored by developmental time point (left) or cell type (right). Post-natal samples include skeletal muscle tissues obtained from patients between the ages of 7 and 42 years**. B)** Proportion of cell types across developmental time points. Data reanalyzed from Xi et al. [3] **C)** Dotplot showing expression of marker genes of human skeletal mesenchymal cells across the identified FN-RMS cell states. **D)** Barplot showing the proportion of RMS cells mapping to cell types found in human skeletal muscle development as defined by Xi et al. [3]. Cell types from human skeletal muscle development were projected onto RMS cells using SingleR based on their most similar developmental time point.

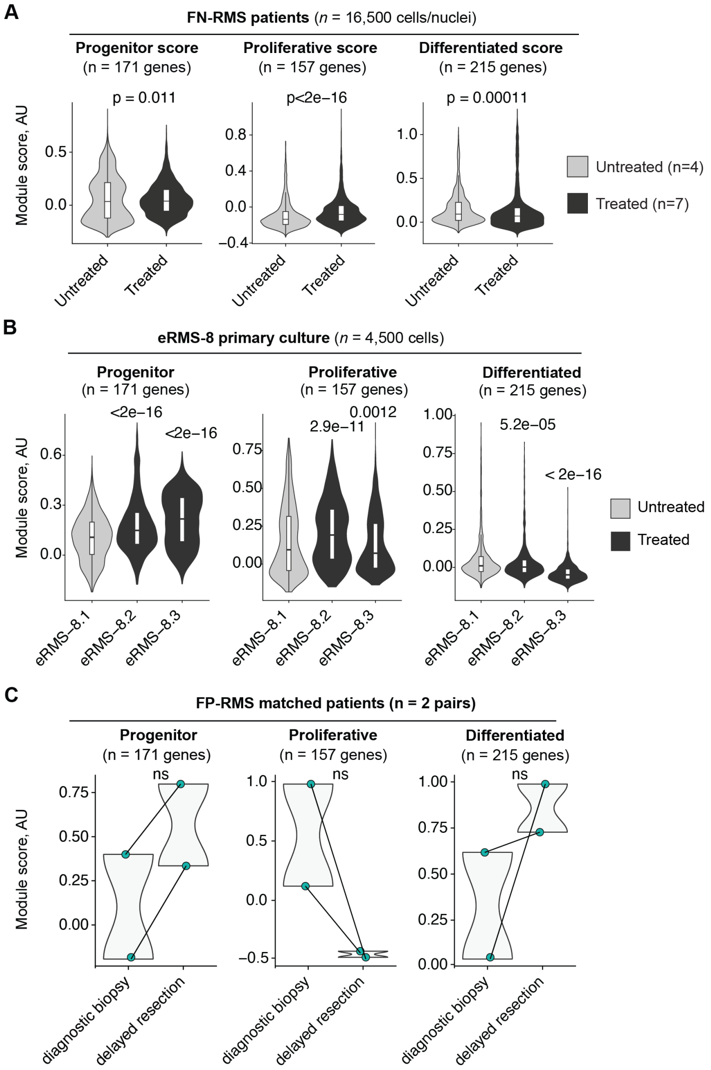

Fig. S6. FN-RMS progenitor cells are increased following chemotherapy treatment. **A)** Metaprogram scores from FN-RMS snRNA-seq datasets assigned across cells/nuclei derived from untreated and treated FN-RMS patient tumors. *P*-values were calculated by Student's T-test. **B**) Comparison of metaprogram scores from eRMS-8 primary culture samples that were obtained from a FN-RMS patient before (eRMS-8.1) and during therapy (eRMS-8.2 and eRMS-8.3). *P*-values were calculated by one-way ANOVA. **C)** RNA sequencing analysis of paired diagnostic and on-therapy resection FP-RMS tumors.

Metaprogram scores calculated using the RMS-atlas signature gene sets are shown. P-values, Wilcoxon signed ranked test.

Reference

1. DeMartino, J., et al., *Single-cell transcriptomics reveals immune suppression and cell states predictive of patient outcomes in rhabdomyosarcoma.* Nat Commun, 2023. **14**(1): p. 3074.
2. Gryder, B.E., et al., *PAX3-FOXO1 Establishes Myogenic Super Enhancers and Confers BET Bromodomain Vulnerability.* Cancer Discov, 2017. **7**(8): p. 884-899.
3. Xi, H., et al., *A Human Skeletal Muscle Atlas Identifies the Trajectories of Stem and Progenitor Cells across Development and from Human Pluripotent Stem Cells.* Cell Stem Cell, 2020. **27**(1): p. 181-185.
