# Supplementary material for: Single cell transcriptomic profiling identifies tumor-acquired and therapy-resistant cell states in pediatric rhabdomyosarcoma
Source: Nat Commun. 2024 Jul 26;15:6307. doi: 10.1038/s41467-024-50527-2 (PMC11282092; doi:10.1038/s41467-024-50527-2)
Supplement: Supplementary file 3 — Description of Additional Supplementary Files [file 41467_2024_50527_MOESM3_ESM.pdf]

Description of Additional Supplementary Files

File Name: Supplementary Data 1

Description: Clinical properties of the RMS samples used for this analysis.

File Name: Supplementary Data 2

Description: Marker genes differentially expressed among the identified Seurat clusters in the integrated RMS atlas (FC>0.3).

File Name: Supplementary Data 3

Description: Gene enrichment analysis of the marker genes differentially expressed among Seurat clusters in the integrated RMS atlas (FC>0.3).

File Name: Supplementary Data 4

Description: Marker genes for the progenitor, proliferating and differentiated cell states from the original publications and this new analysis.

File Name: Supplementary Data 5

Description: Cluster markers identified across the FN-RMS, FP-RMS (PAX3::FOXO1) and FP-RMS (PAX7::FOXO1) RMS subtype-specific datasets. Marker genes differentially expressed among the identified Seurat clusters (FC>0.25).

File Name: Supplementary Data 6

Description: Gene enrichment analysis of the marker genes differentially expressed among clusters in subtype specific analysis of FN-RMS, FP-RMS (PAX3::FOXO1) and FP-RMS (PAX7::FOXO1) RMS (FC>0.25).

File Name: Supplementary Data 7

Description: Frequency of cells across the clusters identified in the FN-RMS, FP-RMS (PAX3::FOXO1) and FP-RMS (PAX7::FOXO1) RMS subtype-specific datasets.

File Name: Supplementary Data 8

Description: Gene markers with predicted membrane localization enriched in the FN-RMS, FP-RMS (PAX3::FOXO1) and FP-RMS (PAX7::FOXO1) tumor subpopulations.

File Name: Supplementary Data 9

Description: Number and fraction of RMS cells mapping to the developmental age and cell types found in normal human muscle.
