# Supplementary material for: Single cell transcriptomic profiling identifies tumor-acquired and therapy-resistant cell states in pediatric rhabdomyosarcoma
Source: Nat Commun. 2024 Jul 26;15:6307. doi: 10.1038/s41467-024-50527-2 (PMC11282092; doi:10.1038/s41467-024-50527-2)
Supplement: Supplementary file 13 — Reporting summary [file 41467_2024_50527_MOESM13_ESM.pdf]

nature portfolio

Corresponding author(s): Marco Wachtel
Beat Schäfer
Anand G. Patel
David Langenau

Last updated by author(s): May 31, 2024

Reporting Summary

Nature Portfolio wishes to improve the reproducibility of the work that we publish. This form provides structure for consistency and transparency in reporting. For further information on Nature Portfolio policies, see our Editorial Policies and the Editorial Policy Checklist.

Statistics

For all statistical analyses, confirm that the following items are present in the figure legend, table legend, main text, or Methods section.

n/a	Confirmed	
☐	☒	The exact sample size (*n*) for each experimental group/condition, given as a discrete number and unit of measurement
☐	☒	A statement on whether measurements were taken from distinct samples or whether the same sample was measured repeatedly
☐	☒	The statistical test(s) used AND whether they are one- or two-sided<br>*Only common tests should be described solely by name; describe more complex techniques in the Methods section.*
☒	☐	A description of all covariates tested
☐	☒	A description of any assumptions or corrections, such as tests of normality and adjustment for multiple comparisons
☐	☒	A full description of the statistical parameters including central tendency (e.g. means) or other basic estimates (e.g. regression coefficient) AND variation (e.g. standard deviation) or associated estimates of uncertainty (e.g. confidence intervals)
☐	☒	For null hypothesis testing, the test statistic (e.g. *F*, *t*, *r*) with confidence intervals, effect sizes, degrees of freedom and *P* value noted<br>*Give P values as exact values whenever suitable.*
☐	☒	For Bayesian analysis, information on the choice of priors and Markov chain Monte Carlo settings
☒	☐	For hierarchical and complex designs, identification of the appropriate level for tests and full reporting of outcomes
☐	☒	Estimates of effect sizes (e.g. Cohen's *d*, Pearson's *r*), indicating how they were calculated

Our web collection on statistics for biologists contains articles on many of the points above.

Software and code

Policy information about availability of computer code

Data collection	No software was used for data collection.
Data analysis	Data pre-processing<br>Those objects were generated using the 10X Genomics Cell Ranger pipeline (version 3.0.1 in Danielli et al ; version 3.1.0 in Wei et al.) to map raw sequencing FASTQ files to the human genome reference (hg19 for patient samples, hg38 for primary cultures) or to both the human hg19 and mouse mm10 references (for PDX samples). Low-quality cells, defined as cells with high mitochondrial ratio (>15% in Danielli et al., >20% in Wei et al.), low expressed gene number (<200 in Danielli et al., <1,000 in Wei et al.), high expressed gene number (>8,000), and PDX cells potentially derived from mice (mouse reads ratio >5% in Wei et al.) were already filtered out. For samples derived from Patel et al., we generated single-cell Seurat objects following the original pipeline. In short, raw sequencing FASTQ files available from GEO were aligned to the human hg19 (for patient samples) or to the combined human hg19 and mouse mm10 references (for PDX samples) using the 10X Genomics Cell Ranger pipeline (version 3.0.0). Low-quality cells, defined as cells with high mitochondrial ratio (>10%), low (<400) or high expressed gene number (>7,000), were filtered out. We further subset each object to keep only malignant tumor cells, defined based on copy-number variation as described in the original publication. For the cell line Rh41, we downloaded the filtered gene-cell matrix available on GEO, that was generated as previously described using the 10X Genomics Cell Ranger pipeline (version 2.0.1) to map raw sequencing FASTQ files to the human hg38 genome reference. Low-quality cells, defined as cells with high mitochondrial ratio (>15%), low (<200) or high expressed gene number (>8,000) were filtered out.<br><br>Merging of single-cell transcriptome data<br>To create the RMS atlas, we first subset each sample to typically n = 1500 randomly selected cells (Table S1), and then merged raw count matrices using Seurat's merge function. This resulted in a total of n = 107,523 cells from n = 72 RMS samples (Table S1). To create the three subtype-specific RMS atlases [(1): FN-RMS (n = 45 samples); (2): PAX3::FOXO1 FP-RMS (n = 15 samples); (3): PAX7::FOXO1 FP-RMS (n = 11

samples)], we merged subtype-specific raw count matrices using Seurat's merge function.

Normalization and data reduction
After merging, we log-normalized the data, selected the top 2,000 variable features downstream analyses, and scaled the gene expression. We then performed principal component analysis (PCA) and, based on elbow plot, selected the top n = 15 principal components (PCs) to consider for downstream analysis. To visualize the cells, we reduced the dimensionality of the datasets using Uniform Manifold Approximation and Projection (UMAP).

Batch correction and clustering
To remove the batch effects from different samples, we integrated the datasets following Seurat's integration pipeline (https://satijalab.org/seurat/archive/v3.0/integration.html), which is based on the identification of anchor cells between pairs of datasets. We first normalized and selected n = 2,000 variable features for downstream integration from each dataset. We then scaled the data and ran PCA on each object. We identified anchors using reciprocal PCA (RPCA), the suggested option for large datasets, and integrated the datasets using the IntegrateData function. We then scaled and centered the gene expression, performed PCA. Based on elbow plot, we then selected the number of PCs to retain for downstream analyses. We built a K-nearest neighbor (KNN) graph, used the Louvain algorithm for clustering the cells (resolution of 0.2-0.3), and visualized the cells using UMAP plots. To identify genes that were enriched within each cluster, we used Seurat's FindAllMarkers function filtering for genes with fold-change >log2(0.25) in the subtype-specific datasets and >log2(0.3) in the integrated dataset and expressed in at least 25% of cells in the cluster.

Annotation of cell clusters
After clustering, we assigned cell states based on the expression of known markers and gene set enrichment analysis. Specifically, we used the marker genes of each cluster as input for Enrichr (https://maayanlab.cloud/Enrichr/), and looked at the GO Biological Process 2023 enriched terms. To annotate and collapse the clusters that contained similar lineages, we used the expression of known markers and gene set enrichment analysis. For example, clusters 6 and 9 of Fig. 1D both expressed high levels of the muscle differentiation markers MYOG, MYL4, MYH3, and were therefore collapsed into one category ('Differentiated') ; clusters 8 and 1 both expressed high levels of the collagen and extracellular matrix genes COL3A1, COL1A1, FN1, and were therefore collapsed into one category ('Progenitor').

RMS cell scoring for meta-programs
Cell-state specific module scoring
To score each cell based on previously identified metaprograms, we selected the gene markers of the original publications as gene inputs (Table S4). We then assigned cell state-specific module scores using the AddModuleScore Seurat's function. This function works by taking an input set of genes and comparing their average relative expression to that of a control set of n = 100 genes randomly sampled. To calculate the consensus progenitor, proliferative, and differentiated marker gene set, we selected cell state markers that were enriched in at least two original publications (Table S4), or in one of the original publications and in the integrated RMS atlas clusters. We then assigned cell state-specific module scores using the AddModuleScore Seurat's function. We defined the muscle lineage score by subtracting the progenitor score from the differentiated score. Unless otherwise specified, cells were scored using the new consensus progenitor, proliferative, and differentiated markers. The datasets were scaled using the ScaleData Seurat's function to center the expression values.

Cell-cycle scoring
After integration, we assigned cell cycle scores using Seurat's CellCycleScoring function, which relies on gene signatures that have been previously shown to characterize S and G2/M cell cycle phases. We distinguished high cycling (S-scores or G2/M scores > 0) from low cycling cells (S-scores < 0 and G2/M scores < 0) based on S and G2/M scores.

Comparison of RMS tumors with single-cell reference data from human development
To infer comparisons between RMS tumors and human skeletal muscle development, we re-analyzed a scRNAseq dataset of human skeletal muscle development (GEO: GSE147457). We downloaded gene expression matrices and their corresponding metadata information for the myogenic subsets derived from embryonic development (1), fetal development (2), juvenile (3) and adult (4) directly from the authors (http://cells.ucsc.edu/?ds=skeletal-muscle). After merging the raw count matrices of the individual datasets, we log-normalized the data, selected the top 2,000 variable features for downstream analyses, and scaled the gene expression. We then performed PCA and, based on elbow plot, selected the top n = 10 PCs for downstream analysis. To visualize the cells, we reduced the dimensionality of the datasets using Uniform Manifold Approximation and Projection (UMAP). To recognize the cell types and developmental time points at which RMS tumors might arise, we used SingleR, a computational framework that takes a dataset with known labels as an input and that transfers them onto a test dataset based on similarity to the reference. Specifically, we projected signatures from the human development dataset onto our combined FN-RMS, PAX3::FOXO1 FP-RMS and PAX7::FOXO1 FP-RMS single cell objects.

Bulk RNA-seq
To score FFPE tissues and orthotopic PDX biopsies for the progenitor, proliferative or differentiated scores, we first created a Seurat object using the already TPM-normalized read count matrix, and log-normalized the count matrix expression values+1. We then scored individual samples for the cell state-specific module scores using the AddModuleScore Seurat's function. We plotted the scores after scaling and centering the expression values using the ScaleData Seurat's function.

Code Availability
The code used to generate the results reported in this manuscript are available through a Github repository [https://github.com/Sara-Danielli/RMS-metadata].

For manuscripts utilizing custom algorithms or software that are central to the research but not yet described in published literature, software must be made available to editors and reviewers. We strongly encourage code deposition in a community repository (e.g. GitHub). See the Nature Portfolio guidelines for submitting code & software for further information.

Data

Policy information about availability of data

All manuscripts must include a data availability statement. This statement should provide the following information, where applicable:

- Accession codes, unique identifiers, or web links for publicly available datasets
- A description of any restrictions on data availability
- For clinical datasets or third party data, please ensure that the statement adheres to our policy

Published single-cell/nucleus RNA-sequencing data were obtained from the Gene Expression Omnibus (GEO): Danielli et al. (GSE218974); Patel et al. (GSE174376); Wei, et al. (GSE195709); Cheng et al. (GSE113660). RNA-sequencing data generated from matched patient samples before and during therapy as well as biopsied FP-RMS orthotopic PDXs are available at the GEO under accession number GSE240287 and GSE240308, respectively. The code used to generate the main results of this manuscript are available at https://github.com/Sara-Danielli/RMS-metadata. The RMS single-cell objects generated in this study have been uploaded on FigShare: https://figshare.com/projects/RMS_consensus_analysis/194417.

Research involving human participants, their data, or biological material

Policy information about studies with human participants or human data. See also policy information about sex, gender (identity/presentation), and sexual orientation and race, ethnicity and racism.

Reporting on sex and gender	This study utilized 72 previously published single-cell/nucleus datasets from 3 studies. Where available, we have compiled and provided gender information from the original studies in Table S1.
Reporting on race, ethnicity, or other socially relevant groupings	No race or ethnicity data were available from the original studies, so that information has not been included in the analysis
Population characteristics	Population characteristics from the combined dataset can be comprehensively reviewed in Table S1. In summary, we collected the following demographic information whenever available: age, gender, site of disease, whether the sample was from a diagnostic or recurrent site, whether the sample was obtained before or after treatment with chemotherapy
Recruitment	All datasets from this study were either previously published, or obtained from retrospective archived tissue. All archived tissue from the pre and mid-treatment analyiss trial were utilized from patients who consented to tissue banking.
Ethics oversight	All data utilized in this dataset were either obtained from previously published datasets (single-cell/nucleus RNA-seq data) or from retrospective analysis of banked tumor tissue. The utilization of banked tissue was overseen by the St. Jude Children's Research Hospital Institutional Review Board.

Note that full information on the approval of the study protocol must also be provided in the manuscript.

Field-specific reporting

Please select the one below that is the best fit for your research. If you are not sure, read the appropriate sections before making your selection.

☒ Life sciences ☐ Behavioural & social sciences ☐ Ecological, evolutionary & environmental sciences

For a reference copy of the document with all sections, see nature.com/documents/nr-reporting-summary-flat.pdf

Life sciences study design

All studies must disclose on these points even when the disclosure is negative.

Sample size	Sample size calculations were not performed for this study, as this study utilized sequencing previously generated from limited patient or patient-derived tissue.
Data exclusions	No data was excluded from this study.
Replication	To ensure consistency of our findings, we compared duplicated xenograft datasets that were inoculated, expanded and processed in separate labs (Figure S1).
Randomization	Not applicable. This study utilized previously generated datasets.
Blinding	Not applicable. This study utilized previously generated datasets.

Reporting for specific materials, systems and methods

April 2023

We require information from authors about some types of materials, experimental systems and methods used in many studies. Here, indicate whether each material, system or method listed is relevant to your study. If you are not sure if a list item applies to your research, read the appropriate section before selecting a response.

Materials & experimental systems

n/a	Involved in the study
☐	☒ Antibodies
☒	☐ Eukaryotic cell lines
☒	☐ Palaeontology and archaeology
☐	☒ Animals and other organisms
☒	☐ Clinical data
☒	☐ Dual use research of concern
☒	☐ Plants

Methods

n/a	Involved in the study
☒	☐ ChIP-seq
☒	☐ Flow cytometry
☒	☐ MRI-based neuroimaging

Antibodies

Antibodies used	Tissues were fixed in 10% neutral buffered formalin, paraffin embedded, sectioned at 4 µm, and mounted onto glass slides (Superfrost Plus; 12-550-15, Thermo Fisher Scientific, Waltham, MA). Slides were then dried for 20 minutes at 60°C, deparaffinized, and stained with hematoxylin and eosin (Richard-Allan Scientific) or used in immunohistochemistry experiments. HE sections were stained and coverslipped using the HistoCore SPECTRA Workstation (Lecia Biosystems). Serial sections were immunolabeled with Synaptophysin (Abcam, ab32127, 1:400) using a Ventana Discovery Ultra autostainer (Roche, Indianapolis, IN) and the following conditions: Heat-induced epitope retrieval, Cell Conditioning Solution ULTRA CC1 (950-224, Roche) for 32 minutes and visualization with DISCOVERY OmniMap anti-Rb HRP (760-4311, Roche), Hematoxylin II (790-2208, Roche), and Bluing reagent (760-2021, Roche). MYOGENIN staining (Abcam, ab1835, 1:150) was performed on a Ventana Discovery Ultra autostainer (Roche, Indianapolis, IN) using the following conditions: Heat-induced epitope retrieval, Cell Conditioning Solution ULTRA CC2 (950-223, Roche) for 60 minutes and visualization with DISCOVERY OmniMap anti-Rb HRP (760-4311, Roche), Hematoxylin II (790-2208, Roche), and Bluing reagent (760-2021, Roche). Whole slide images to a 20x scalable magnification were created using a PANNORAMIC 250 Flash III digital slide scanner (3DHISTECH Ltd, Budapest, Hungary). Images were taken using the HALO v3.6.4134.137 software program (Indica Labs) and analyzed using HALO v3.2.1851.354 and the Area Quantification FL v2.3 algorithm to determine the area of immunoreactivity for each marker (all Indica Labs, Albuquerque, NM). Visual interpretations of immunohistochemical staining were conducted by a board-certified veterinary pathologist and in a manner that was blinded to the experimental condition of each mouse and compared with image analysis findings.
Validation	Antibodies were validated using known positive (RMS) or negative (neuroblastoma) controls.

Animals and other research organisms

Policy information about studies involving animals; ARRIVE guidelines recommended for reporting animal research, and Sex and Gender in Research

Laboratory animals	Immunodeficient mice
Wild animals	Not applicable.
Reporting on sex	All immunodeficient mice used in this study were female sex, as is standard practice for passaging of patient-derived xenografts.
Field-collected samples	Not applicable
Ethics oversight	Animal experiments were performed after approval by the St. Jude Institutional Animal Care Use Committtee. Immunodeficient mice were housed according to IACUC standards using barrier conditions and isolation cages to minimize pathogen exposure. The housing facility operates with an alternating light schedule (12 hours on, 12 hours off) and has a dedicated isolated ventilation system. All mice were fed and provided water ad libitum.

Note that full information on the approval of the study protocol must also be provided in the manuscript.

Plants

Seed stocks	N/A
Novel plant genotypes	N/A
Authentication	N/A
